# The Value of Hysterosalpingography following Medical Treatment with Methotrexate for Ectopic Pregnancy

**DOI:** 10.1155/2011/547946

**Published:** 2011-09-27

**Authors:** Emma Garcia Grau, Miguel Ángel Checa Vizcaíno, Mário Oliveira, Judith Lleberia Juanós, Ramon Carreras Collado, Yolanda Canet Estevez

**Affiliations:** ^1^Department of Obstetrics and Gynecology, Corporació Sanitària Parc Taulí, 08208 Sabadell, Barcelona, Spain; ^2^Department of Obstetrics and Gynecology, Hospital Universitari del Mar, Universitat Autònoma de Barcelona, 08003 Barcelona, Spain; ^3^Life and Health Sciences Research Institute (ICVS), School of Health Sciences, University of Minho, 4710-057 Braga, Portugal

## Abstract

After an ectopic pregnancy (EP) fertility decreases, mostly due to tubal factor. Hysterosalpingography (HSG) is the most cost-effective tool for tubal patency assessment. *Objective*. To evaluate the usefulness of a HSG after a medical treatment for an EP, in order to counsel women on the most appropriate way to conceive future pregnancies. *Methods*. Between 1998 and 2008, 144 patients were submitted to medical treatment for an EP and performed HSG 3 months after the event. *Results*. 72.2% of normal HSG, 18.8% with unilateral obstruction, 6.3% tubal patency with defect, and 2.8% bilateral obstruction. *Conclusion*. Routine HSG following medical treatment for an EP does not seem necessary, as it does not change the initial management in 97.2% of the cases, but might be considered in selected risk cases, permitting timely referral of patients to in vitro fertilization.

## 1. Introduction

Ectopic pregnancy (EP) is defined as the abnormal implantation of the blastocyst outside the uterine endometrium. It affects 2% of all pregnancies and is associated with significant maternal morbid mortality, being the main cause of maternal death during the first trimester of gestation [1]. The most common site of implantation is the Fallopian tube (in the fimbrial end, ampullary section, or isthmus) but can also be interstitial, ovarian, abdominal and cervical, or on the site of a previous cesarean section scar [2].

Females with abnormal Fallopian tubes are at increased risk of developing an EP [3]. Thus, the risk factors for developing EP include pelvic inflammatory disease (PID), history of EP, infertility, history of pelvic or abdominal surgery, endometriosis, sexually transmitted diseases, previous tubal surgery (namely tubal sterilization), smoking, older maternal age, and in uterus exposure to diethylstilbestrol.

Some of these conditions result in scar tissue at the Fallopian tubes and may thus prevent the fertilized egg to reach the uterine cavity. 

Despite the fact that the treatment is classically surgical, early diagnosis allows the implementation of a medical treatment [4], thus avoiding the surgery-associated morbidity and maintaining the patient's anatomy.

Several medical treatments have been used such as prostaglandins, dactinomycin, etoposide, hyperosmolar glucose, anti-hCG antibodies, potassium chloride, or mifepristone. However, the treatment with methotrexate (MTX) has shown better results and is presently considered the first option for medical therapy [5].

MTX is chemotherapeutic drug, antagonist of folic acid and acts as an antimetabolite, by combining with the enzyme tetrahydrofolate reductase. It inhibits the synthesis of puric and pyrimidic bases, essential for the formation of DNA and RNA. Its action is exerted on cells with fast replication, including the trophoblast [6].

The medical treatment with methotrexate may be applied systemically (intramuscular administration of MTX) or by direct injection inside the gestational sac, whether guided by ultrasound or by a laparoscopic approach.

MTX destroys cells in fast replication (in this case the trophoblast). However, this process may result in residual lesion to the Fallopian tube. Fertility after a conservative treatment for an EP can be attested indirectly through a hysterosalpingography (HSG) or directly by diagnosing a new gestation [7].

HSG is the radiographic evaluation of the uterine cavity and fallopian tubes after the administration of a radio-opaque medium through the cervical canal. The first HSG was performed in 1910 and was considered to be the first special radiologic procedure. A properly performed HSG can detect the contour of the uterine cavity and the width of the cervical canal. Further contrast medium injection will outline the cornua isthmic and ampullary portions of the tubes and will show the degree of spillage.

It has a sensibility of 0.65 (CI 95% 0.5–0.78) and a specificity of 0.83 (CI 95% 0.77–0.88) [8]. Thus, given the high specificity of the HSG, in case of diagnosis of tubal obstruction, there is a high probability that it really exists, while the observation of tubal permeability shown by the exam does not exclude tubal pathology, since it does not assess its function.

On the other side, the HSG has a high reproducibility both intra- and interobservers, mainly in cases of proximal obstructions [9]. 

In addition, HSG is a safe and inexpensive procedure [10], being the most cost-effective method in the study of the Fallopian tubes [11].

Evidence shows that fertility decreases following an EP [12]. Future fertility in these patients is dependent on several factors, including age, history of infertility, history of previous EP, tubal rupture, and contralateral tubal lesion [13]. Thus, it seems reasonable to assess tubal permeability following a medical treatment of an EP in those women who are willing to have future progeny.

The aim of this study is to assess the usefulness of HSG following medical treatment of EP in order to counsel patients willing to have future gestations about the most appropriate method of conception. The secondary aims of the study are to evaluate the clinical effectiveness of the different modalities of treatment and fertility rates following a medical treatment of EP.

## 2. Materials and Methods

### 2.1. Study Design and Participants

The Ethics Committee of the Corporació Sanitària Parc Taulí approved this study. An observational retrospective study was performed, including all women submitted to HSG following conservative management of an EP at the Institution between 1998 and 2008.

### 2.2. Treatments for Ectopic Pregnancy

Indications for conservative treatment with methotrexate (MTX) were [14] hemodynamic stability without evidence of active bleeding or hemoperitoneum, willingness of having future progeny, possibility of followup, absence of contraindications for MTX, gestational sac with maximum diameter of 4 cm, and no embryonic cardiac motion.

The maximum initial beta human chorionic gonadotrophin (*β*-HCG) serum level considered for intramuscular systemic treatment was 3000 mUI/mL and 10000 mUI/mL for intrasacular injection. Additionally, in case of intrasacular ultrasonographically guided treatment, gestational sac should be accessible to ultrasonography guidance.

Contraindications for treatment with MTX included [5] intrauterine gestation, immunodeficiency, moderate to severe anemia, leucopenia or thrombocytopenia, known MTX sensitivity, acute respiratory disease, active peptic ulcer disease, major hepatic or renal impairment, and breastfeeding.

MTX was administered as a standard dose of 50 mg for intrasacular injection and 50 mg/m^2^ of body surface area (BSA) for systemic intramuscular treatment. The formula used for assessment of BSA in square meters was: [height (cm) + weight (Kg) − 60]/100.

### 2.3. Hysterosalpingography

A pregnancy test was ruled out before the HSG and profilaxis with doxycycline was recommended to all patients. Because patients may experience cramping during the HSG, women were advised to take a nonsteroidal anti-inflammatory drug one hour prior to the procedure. There were two contraindications for HSG: pregnancy and active pelvic infection.

The procedure was performed with a sterile technique, with a single-tooth tenaculum applied to align the cervical canal and uterine cavity. Approximately 10 mL of water-soluble contrast medium was injected through a cannula. Fluoroscopic examination was performed during the injection with patient repositioning as necessary.

HSG was performed in immediate postmenstrual phase, 3 months after the medical treatment for the EP, when the image of an extraovarian adnexal mass disappears at transvaginal ultrasound. 

The examination was performed, read, and interpreted by a radiologist. The radiographic findings were scored as: normal, unilateral patency, patency but with defects (namely a no obstructive hydrosalpinx), and bilateral obstruction.

### 2.4. Data Collection

Data were gathered concerning patient demographic characteristics (age, parity, risk factors for EP) and details of the occurrence (laterality of EP, type of treatment, outcome of treatment, and HSG findings). In March 2009, a phone query was performed, and data concerning later gestations and mode of conception, whether spontaneous or through medically assisted reproduction, were collected.

### 2.5. Statistical Analysis

All used tests were bilateral. The level of statistical significance was considered for   *P* < 0.05. Statistical analysis was performed using the software PASW Statistics version 18.0.0 (SPSS Inc., IBM Corporation, Chicago. Il, USA). 

Quantitative variables were described through averages and standard deviations. Categorical variables were described by frequencies. 

Distribution of probabilities for quantitative variables was tested for normality by Kolmogorov-Smirnov test. Differences between groups were analyzed by Student's *t*-test for normal quantitative variables. The nonparametric Mann-Whitney *U* test was used in case of nonnormality; differences between proportions were analyzed with the Pearson's chi-square test or Fischer's exact test.

## 3. Results

### 3.1. Patients' Characteristics and Treatment Effectiveness

HSG was performed in 144 women, following medical treatment of EP. Patients*'* average age was 30.27 years (range 16–40). Risk factors for EP were absent in 42, 76% of the population. Intramuscular treatment was performed in 84 cases (58.3%) and intrasacular in 60 (41.7%). The initial treatment was effective in 91% of the situations.  Table 1 shows the effectiveness of the different types of treatment. We failed to reveal statistical significant differences between treatment modality (*P* = 0.562). 

Initial treatment failed in 13 cases (9%), 6 following intramuscular MTX, 2 following ultrasonography-guided MTX intrasacular injection, and 5 after laparoscopic MTX intrasacular injection. A second treatment was offered to these 13 patients: 10 were treated with intramuscular MTX and 3 with salpingectomy. One intramuscular MTX treatment failed and was managed by salpingectomy. Thus, only 4 out of 144 patients required ablative surgery, with the remaining being successfully managed by medical treatment.

### 3.2. Findings on Hysterosalpingography and Subsequent Fertility

Among the 144 HSG performed in women submitted to medical treatment for EP, 104 (72.2%) were normal, 27 (18.8%) displayed unilateral obstruction, 4 (2.8%) bilateral obstruction, and 9 (6.3%) tubal patency, but with defect.  Table 2 shows the findings on HSG according to the different types of treatment. Significant differences on HSG findings were depending on the therapeutic modality (*P* = 0.025), being the intramuscular treatment the one with a greater number of normal HSG. Similar findings were observed in the group of patients that required a second treatment (*P* = 0.044).

 No significant differences were found in the HSG findings when comparing patients submitted to a single or an additional treatment (*P* = 0.124).

23 patients (15.97%) were lost to follow up. Among the remaining 121 who received medical treatment for EP, 83.5% accomplished a subsequent gestation, 78.5% without medically assisted reproduction. Moreover, as shown in Table 3, the type of treatment did not significantly affect the pregnancy rate (*P* = 0.127). No differences were found in the pregnancy rate in the group of patients submitted to an additional treatment (*P* = 0.4).

In face of an unchanged HSG, 90.6% of the women achieved a later gestation. However, findings of unilateral obstruction resulted in a decrease of the pregnancy rate to 76% and to 62.5% in case of tubal patency with alteration (Table 4). These differences were statistically significant (*P* < 0.0005). 

All patients in the group of bilateral obstruction (3 patients) failed to attain a later gestation, with two presently in process of in vitro fertilization. Among them, one had previous history of an additional EP, other had a cornual pregnancy following several myomectomies treated with ultrasonography-guided intrasacular MTX injection, and the other a torpid evolution. Subsequent spontaneous pregnancy rate was also significantly affected by the HSG findings (*P* = 0.004).

## 4. Discussion

Our data show that findings on HSG were different according to the therapeutic approach, with the intramuscular treatment carrying an increased ratio of normal exams, when compared to the intrasacular injection. Two reasons could explain these findings: the inclusion criteria for intrasacular treatment allow higher initial levels of *β*-HCG. The increase in *β*-HCG levels is related to an enhancement in tubal obstruction risk, probably because in patients with high levels of *β*-HCG there is more invasion of the trophoblast tissue at the serosa of the tube, which increases the damage [15]. Additionally, the intrasacular treatment requires direct tubal puncture, possibly injuring the Fallopian tube with consequent increased scarring and additional peritubal peritoneal adhesions.

Nevertheless, it must be highlighted that data is lacking on the basis of the tubal defects identified after an EP, whether they were a consequence of the treatment or were already present and were thus the cause of an abnormal implantation.

The subsequent pregnancy rate was also significantly influenced by the findings on the HSG, being higher in the patients with normal HSG. Moreover, an increased proportion of spontaneous gestations was observed in these subjects. Mol et al. reported fecundity rate ratios of one-sided and two-sided abnormalities detected with HSG of 0.93 and 0.35, respectively [16]. 

Despite the obvious disadvantages of being a retrospective study, in light of the present results, it seems reasonable to consider that performing a HSG following medical treatment of an EP could provide prognostic information regarding future attempts to accomplish gestation.

Conversely, in 97.2% of the patients the HSG findings would be unlikely to affect the initial conduct that would probably be expectant, including the group of fertile women exhibiting unilateral tubal obstruction or tubal patency with alteration. Furthermore, only the 2.8% of women diagnosed bilateral tubal obstruction would probably benefit from HSG, being recommended in vitro fertilization. As previously stated, these patients presented history of previous tubal pathology or difficult management of EP. Therefore, it could be considered the benefit of performing HSG in such patients, in whom a prompt diagnosis of bilateral obstruction would result in adequate counseling towards in vitro fertilization, devoid of delay.

The medical treatment with MTX for EP is effective and safe: 91% success on a first treatment (considering both intramuscular and intrasacular administration) without any relevant complication. The Practice Committee of the American Society for Reproductive Medicine published in 2008 that MTX treatment was successful in 78%–96% of selected patients [17]. It seems clear that in order to achieve comparable good results, compliance with patient selection criteria is essential.

Fertility following medical treatment was noticeably high (83.5%), with 78.5% spontaneous pregnancies. Nevertheless, it must be considered that not all patients sought pregnancy after treatment; thus, fertility rates could even achieve higher levels if only women seeking further gestation were considered or if no limitations existed on the access to medically assisted reproduction

## 5. Conclusions

Findings on HSG carry a prognostic value when considering future fertility, following medical treatment of EP. However, initial management is affected only in 2.8% of the women. Further studies should be undertaken in order to confirm the present results that suggest that routine HSG following medical treatment for an EP does not seem necessary but might be considered in selected risk cases, permitting timely referral of patients to in vitro fertilization

## Figures and Tables

**Table 1:** Effectiveness of the different medical treatment modalities. Results are expressed as frequencies and percentage (*P* = 0.562).

			Success of treatment	
			Yes	No
Treatment	Intramuscular	Total	78	6
		%	92.9%	7.1%
	Ultrasonography-guided intrasacular injection	Total	20	2
		%	90.9%	9.1%
	Laparoscopic intrasacular injection	Total	33	5
		%	86.8%	13.2%

Total		Total	131	13
		%	91%	9%

**Table 2:** Findings on hysterosalpingography according to initial treatment modality for ectopic pregnancy. Results are expressed as frequencies and percentage (*P* = 0.025).

			Findings on hysterosalpingography			
			Normal	Unilateral patency	Bilateral obstruction	Patency with alteration
Treatment	Intramuscular	Total	69	11	1	3
		%	82.1%	13.1%	1.2%	3.6%
	Ultrasonography-guided intrasacular injection	Total	14	5	2	1
		%	63.6%	22.7%	9.1%	4.5%
	Laparoscopic intrasacular injection	Total	21	11	1	5
		%	55.3%	28.9%	2.6%	13.2%

Total		Total	104	27	4	9
		%	72.2%	18.8%	2.8%	6.3%

**Table 3:** Pregnancy rate according to treatment administered for ectopic pregnancy. Results are expressed as frequencies and percentage (*P* = 0.127).

			Subsequent pregnancy	
			Yes	No
Treatment	Intramuscular	Total	62	8
		%	88.6%	11.4%
	Ultrasonography-guided intrasacular injection	Total	14	6
		%	70%	30%
	Laparoscopic intrasacular injection	Total	25	6
		%	80.6%	19.4%

Total		Total	20	101
		%	16.5%	83.5%

**Table 4:** Accomplishment of subsequent pregnancy according to the findings on hysterosalpingography (HSG). Results are expressed as frequencies and percentage (*P* < 0.0005).

			Subsequent pregnancy	
			Yes	No
Findings on HSG	Normal	Total	77	8
		%	90.6%	9.4%
	Unilateral patency	Total	19	6
		%	76.0%	24.0%
	Bilateral obstruction	Total	0	3
		%	0%	100%
	Patency with alteration	Total	5	3
		%	62.5%	37.5%

Total		Total	20	101
		%	16.5%	83.5%
